# VMAT2 availability in Parkinson’s disease with probable REM sleep behaviour disorder

**DOI:** 10.1186/s13041-021-00875-7

**Published:** 2021-11-10

**Authors:** Mikaeel Valli, Sang Soo Cho, Carme Uribe, Mario Masellis, Robert Chen, Alexander Mihaescu, Antonio P. Strafella

**Affiliations:** 1 Brain Health Imaging Centre, Campbell Family Mental Health Research Institute, Centre for Addiction and Mental Health, University of Toronto, Toronto, ON Canada; 2 Division of Brain, Imaging and Behaviour – Systems Neuroscience, Krembil Brain Institute, UHN, University of Toronto, Toronto, ON Canada; 3 Institute of Medical Science, University of Toronto, Toronto, ON Canada; 4 Department of Brain and Cognitive Science, Seoul National University, Seoul, Republic of Korea; 5 Hurvitz Brain Sciences Program, Sunnybrook Research Institute, Toronto, ON Canada; 6 Division of Neurology, Department of Medicine, University of Toronto, Toronto, ON Canada; 7 Edmond J. Safra Parkinson Disease Program & Morton and Gloria Shulman Movement Disorder Unit, Neurology Division, Dept. of Medicine, Toronto Western Hospital, UHN, University of Toronto, Toronto, ON Canada

**Keywords:** Parkinson’s disease, REM sleep behaviour disorder, Positron emission tomography, VMAT2, [^11^C]DTBZ

## Abstract

REM sleep behaviour disorder (RBD) can be an early non-motor symptom of Parkinson’s disease (PD) with pathology involving mainly the pontine nuclei. Beyond the brainstem, it is unclear if RBD patients comorbid with PD have more affected striatal dopamine denervation compared to PD patients unaffected by RBD (PD-RBD−). To elucidate this, we evaluated the availability of vesicular monoamine transporter 2 (VMAT2), an index of nigrostriatal dopamine innervation, in 15 PD patients with probable RBD (PD-RBD+), 15 PD-RBD−, and 15 age-matched healthy controls (HC) using [^11^C]DTBZ PET imaging. This technique measured VMAT2 availability within striatal regions of interest (ROI). A mixed effect model was used to compare the radioligand binding of VMAT2 between the three groups for each striatal ROI, while co-varying for sex, cognitive function and depression scores. Multiple regressions were also computed to predict clinical measures from group condition and VMAT2 binding within all ROIs explored. We observed a significant main effect of group condition on VMAT2 availability within the caudate, putamen, ventral striatum, globus pallidus, substantia nigra, and subthalamus. Specifically, our results revealed that PD-RBD+ had lower VMAT2 availability compared to HC in all these regions except for the subthalamus and substantia nigra, while PD-RBD− was significantly lower than HC in all these regions. PD-RBD− showed a negative relationship between motor severity and VMAT2 availability within the left caudate. Our findings reflect that both PD patient subgroups had similar denervation within the nigrostriatal pathway. There were no significant interactions detected between radioligand binding and clinical scores in PD-RBD+. Taken together, VMAT2 and striatal dopamine denervation in general may not be a significant contributor to the pathophysiology of RBD in PD patients. Future studies are encouraged to explore other underlying neural chemistry mechanisms contributing to RBD in PD patients.

## Introduction

Rapid eye movement (REM) sleep behaviour disorder (RBD) is a parasomnia characterized by the loss of normal skeletal muscle atonia during REM sleep [1]. This results in dream enacting behaviours that are often violent or aggressive in nature. Increasing evidence shows that the RBD pathology involves the pontine nuclei within the brainstem [2]. Studies have further shown a link between idiopathic RBD and alpha-synucleinopathies such as Parkinson’s disease (PD) [3, 4]. RBD is one of the hallmark prodromal features that manifests as early as 20 years before official PD diagnosis in many patients [5]. The risk estimate of developing PD is 33% at 5 years since RBD diagnosis. This risk sharply increases to 91% at 14 years [6]. This underscores the importance of RBD in PD pathology as it is a strong clinical predictor of PD and is co-morbid in nearly half of the PD patient population [6].

At the time of PD diagnosis, there is already advanced degeneration within the nigrostriatal dopaminergic system, with estimates of 50–70% dopaminergic terminal loss within the putamen based on a post-mortem study [7] and in vivo neuroimaging of early-stage PD patients with unilateral motor impairment [8]. In the prodromal phase between the time of RBD diagnosis and clinical manifestation of PD, there are multiple neuroimaging studies that show presynaptic striatal degeneration, particularly by indexing the dopamine transporter (DAT) density. DAT is a symporter primarily found on the presynaptic terminals of nigrostriatal neurons. It carries the function of clearing released dopamine from the synaptic cleft back into the presynaptic terminals—thereby modulating dopaminergic transmission [9]. Neuroimaging studies report consistent striatal DAT depletion in 20–40% of polysomnography confirmed idiopathic RBD patients (i.e., without PD) relative to healthy controls, particularly within the putamen [6, 10–13]. The decline of striatal DAT has been demonstrated from healthy controls to sub-clinical RBD (REM sleep without atonia on polysomnography, but without abnormal nocturnal behaviours) to manifest RBD to PD [14, 15]. This worsening pattern continues in a subset of PD patients with probable RBD where they show greater DAT depletion in the caudate and putamen compared to PD patients without probable RBD [16, 17]. Consistently, another study by Arnaldi et al. (2015) showed DAT levels in the putamen progressively decreased from idiopathic RBD to PD without probable RBD to PD with probable RBD. Taken together, these studies implicated that DAT imaging may play a contributory role in detection of RBD and in PD patients with probable RBD.

In addition to measuring DAT density as an index for presynaptic dopaminergic integrity, quantification of the type 2 vesicular monoamine transporter (VMAT2) is another approach. VMAT2 is an integral membrane protein that is responsible for shuttling monoamine neurotransmitters including dopamine from the cytosol to the synaptic vesicles [9]. Evidence shows that VMAT2 is sensitive to changes in vesicular dopamine concentration [18]. However, its binding site was shown to be less sensitive to changes induced by medication or compensatory mechanisms associated with the loss of dopaminergic neurons relative to DAT [19]. Hence, quantifying VMAT2 levels allow for a more accurate measurement of the dopaminergic terminal integrity compared to measuring DAT levels [20]. VMAT2 can be measured with [^11^C]-dihydrotetrabenazine ([^11^C]DTBZ) or [^18^F]AV-133 and both are reliable for in vivo imaging of VMAT2 density and distribution within the basal ganglia [9]. A recent study using [^18^F]AV-133 showed that probable RBD patients had reduced VMAT2 levels within the caudate nuclei and putamen relative to healthy controls [21]. Similar results were shown in a smaller older study using [^11^C]DTBZ where the authors observed reduced VMAT2 availability in the posterior putamen in polysomnography confirmed RBD patients in relation to healthy controls [22]. As presented here, there are limited studies that focused on VMAT2 imaging, but these investigations indicate that VMAT2 imaging may also have a certain role in detecting RBDs.

In summary, while the literature seems to suggest an association of RBD with the presynaptic dopaminergic system, it remains unclear if PD patients with RBD have more extensive striatal dopamine denervation than PD patients without RBD. Furthermore, the characterization of VMAT2 has been rarely explored—especially in PD patients with RBD. By examining VMAT2 as an in vivo molecular target with its unique properties, we aim to elucidate VMAT2 levels in PD patients who are comorbid with probable RBD. We achieved this by using [^11^C]DTBZ PET imaging in PD patients with probable RBD and in PD patients without probable RBD, while having age-matched healthy controls. We hypothesized that PD patients with probable RBD to show more extensive dopamine denervation pathology, that is reduced VMAT2 availability, in striatal regions including the caudate, putamen, and ventral striatum relative to PD patients without probable RBD and healthy controls. This hypothesis would be consistent with reductions of the DAT observed in the striatal regions reported in PD patients with probable RBD.

## Methods

### Participants

A total of 45 participants were enrolled in this study: 15 PD patients without probable RBD, 15 PD patients with probable RBD, and 15 age-matched healthy controls (Table 1). Some of the imaging, demographic, cognitive and psychological data from these participants have been reported previously [23, 24]. Both PD patient groups were diagnosed based on the UK Parkinson Disease Society Brain Bank criteria. All participants had no evidence of other neurological or psychiatric conditions or any other medical conditions that precluded them from the PET and MR imaging. The severity of PD motor symptoms was tested through the Hoehn and Yahr Scale and the United Parkinson Disease Rating Scale (UPDRS-III) while patients were on medication. Levodopa equivalent daily dose (LEDD) calculation for each patient has been previously described by Evans et al. [25]. All participants were age-matched; and PD patients were matched for disease severity based on UPDRS-III score and LEDD.

**Table 1:** Demographic, behavioural, clinical and PET imaging characteristics of participants

	HC	PD-RBD−	PD-RBD+	*p* value
*N* (M:F)	**15** (3:12)	**15** (8:7)	**15** (10:5)	0.03^a^
Age [years] ± SD (range)	67.1 ± 5.14 (58–79)	70.7 ± 5.67 (60–80)	68.1 ± 6.48 (56–80)	0.23
BDI ± SD	2.33 ± 1.29	3.00 ± 1.36	5.00 ± 4.32	0.03
MoCA ± SD	27.6 ± 2.13	24.93 ± 2.93	23.87 ± 3.24	0.002
Disease duration [years] ± SD	**–**	7.20 ± 4.49	6.76 ± 3.67	0.77
UPDRS-III ± SD	**–**	28.53 ± 17.18	23.87 ± 10.84	0.38
Hoehn and Yahr Score ± SD	**–**	2.20 ± 0.41	2.13 ± 0.39	0.68
LEDD [mg] ± SD	**–**	701.70 ± 522.04	723.45 ± 410.75	0.90
[^11^C]DTBZ dose [mCi] ± SD	9.54 ± 0.87	9.33 ± 0.59	9.72 ± 0.45	0.29
[^11^C]DTBZ mass [μg] ± SD	1.84 ± 1.78	1.28 ± 0.53	1.74 ± 1.31	0.48
[^11^C]DTBZ specific activity [mCi/μmol] ± SD	2529.77 ± 1312.82	2658.75 ± 995.19	2319.78 ± 816.03	0.68

BDI, Beck Depression Inventory; HC, healthy controls; LEDD, levodopa equivalent daily dose (calculated according to Evans et al. [25]); MoCA, Montreal Cognitive Assessment; PD-RBD+, PD patients with probable RBD; PD-RBD−, PD patients without probable RBD; UPDRS-III, Unified Parkinson’s Disease Rating Scale III

^a^Pearson Chi-Square

PD patients were screened for RBD as part of their routine neurology clinic visits. This screening was done prior to any imaging data collection, including patients reported previously by our group [23, 24]. Identification of probable RBD symptoms was completed through using the informant-based response to the first question on the Mayo Sleep Questionnaire: “Have you ever seen the patient appear to act out his/her dreams while sleeping.” Patients were classified as clinically probable RBD if the patients’ sleeping partner answered yes to this question [26]. This singular question has been validated against polysomnography, with a sensitivity of 98% and specificity of 74%, in a multicenter prospective cohort study of healthy older adults and suspected neurodegenerative disease [26, 27].

In order to prepare for the PET scan, PD patients performed an overnight 12-h withdrawal from anti-parkinsonian medication to minimize the effect of medication during the scans while maintaining patient comfort and functioning [28]. To prevent excessive fatigue, the PET and structural MRI scans were completed on separate days. Montreal Cognitive Assessment (MoCA; [29]) and Beck Depression Inventory (BDI; [30] were obtained on all participants to assess general cognitive capabilities and depression levels, respectively. All participants provided informed written consent prior to beginning any imaging study procedures which were approved by the research ethics committees for the Centre of Addictions and Mental Health and the University Health Network of the University of Toronto.

### Imaging acquisition

The preparation of the [^11^C]DTBZ radioligand was described previously [31]. PET scans were collected using a three-dimensional (3D) high resolution research tomograph (HRRT) scanner (Siemens, Knoxville, TN). This equipment allows the measurement of radioactivity in 207 brain slices, with a thickness of 1.22 mm each [23]. The detectors of the HRRT are a lutetium oxyorthosilicate/lutetium–yttrium oxyorthosilicate phoswich, with each crystal element measuring 2 × 2 × 10 mm^3^. To minimize head motion, a customized thermoplastic facemask was provided to each participant prior to the HRRT PET scan, and the facemask was secured through the head-fixation system (Tru-Scan Imaging, Annapolis). After securing participants within the PET scanner, a transmission scan was first completed using a single photon point source, ^137^Cs (t_1/2_ = 30.2 years, E_γ_ = 662 keV), which had a duration of 6 min and 9 s. This transmission scan was immediately followed by the acquisition of the emission scan to correct for attenuation (where frame durations were: 1 × background; 15 frames × 60 s; and 15 frames × 300 s). Subsequently, the [^11^C]DTBZ radioligand was injected as a bolus into an intravenous line placed in the antecubital vein. Emission data were collected in list mode for 60 min while subjects were at rest.

The emission list mode data were re-binned into a series of 3D sinograms. The 3D sinograms were gap filled, scatter corrected and Fourier re-binned into 2-dimensional (2D) sinograms. The images were reconstructed from the 2D sinograms using a 2D filtered-back projection algorithm. The reconstructed images had 256 × 256 × 207 cubic voxels that measured 1.22 × 1.22 × 1.22 mm^3^. The dynamic images were then reconstructed into 17 frames. The first frame was variable as it was dependent on the time between the start of acquisition and the introduction of the [^11^C]DTBZ radioligand in the tomograph field of view. The following frames were defined as: 1 ×  ≥ 22 s, 4 × 60 s, 3 × 120 s, 8 × 300 s, and 1 × 600 s.

To provide anatomical reference for the parametric PET image analysis and to rule out structural lesions, a whole-brain T1-weighted MR image was acquired from each participant using GE Signa HD × MRI system (GE Discovery MR750 3 T; T1-weighted images, fast spoiled gradient echo with repletion time = 6.7 ms, echo time = 3.0 ms, flip angle = 8 mm, slice thickness = 1 mm, number of excitations = 1, and matrix size = 256 × 192).

### Imaging analysis

Image preprocessing was completed using an in-house software, Regions of Mental Interest (ROMI; [32]). This software was used to obtain the time activity curve (TAC) for the reference region—the occipital lobe—for all participants. ROMI used Statistical Parametric Mapping (SPM8, Welcome Department of Imaging Neuroscience, London, UK), where each participant’s MR image was used to nonlinearly transform a standardized brain template (International Consortium for Brain Mapping/Montreal Neurological Institute 152 MRI) with predefined regions of interests (ROIs). The individual ROI template underwent further refinement based on the gray matter probability of the segmented MRI. The refinement of each individual’s ROIs were then aligned and resliced using a normalized mutual information algorithm [33] to match the individuals PET scan. Subsequently, the TAC for the occipital lobe was obtained from the dynamic [^11^C]DTBZ PET image in the native space.

Upon the completion of the pre-registration procedure with ROMI, [^11^C]DTBZ PET parametric non-displaceable binding potential (BP_ND_) maps were generated in the native PET space with simplified reference tissue model [34] using the occipital cortex TAC value as reference region (obtained through ROMI). This was completed using Receptor Parametric Mapping software (RPM; [35]) within MATLAB R2015a (version 8.5.0.197613; MathWorks). Using SPM12 (version 7487) within MATLAB, the parametric BP_ND_ images were transformed into standardized stereotaxic space using each participants’ individual MRI. These normalized images were then smoothed with a Gaussian function at 8 mm full width half-maximum.

The ROIs we examined were caudate, putamen, internal globus pallidus, external globus pallidus, substantia nigra, and subthalamus. These ROIs were obtained from the WFU-PickAtlas toolbox (http://www.fmri.wfubmc.edu/cms/software). In addition, we examined associative striatum, motor striatum, and ventral striatum—which were delineated according to previously specified criteria [36]. These ROIs were transformed into a parametric [^11^C]DTBZ PET BP_ND_ map, and the BP_ND_ values were extracted using MATLAB based REX toolbox (http://web.mit.edu/swg/software.htm). We used the BP_ND_ values obtained through REX for each ROI for statistical analysis.

### Statistical analysis

Demographic characteristics were tested for differences between the three groups (i.e., healthy controls, PD patients without probable RBD, and PD patients with probable RBD) using ANOVA. Specifically, we performed ANOVA and Bonferroni post-hoc testing on age, MoCA score, BDI, UPDRS-III score, Hoehn and Yahr score, LEDD amount, quality and quantity of injected radioligand across all three participant groups. To assess for differences in sex proportions between groups, a chi-squared analysis was performed. Statistical outliers was investigated using the interquartile range method [37].

Mixed effects model was used to compare the extracted [^11^C]DTBZ BP_ND_ between the three groups for each ROI. The fixed factors within the model were group (i.e., healthy controls, PD patients without probable RBD, and PD patients with probable RBD) and side (i.e., left vs. right ROI); the participants were kept as the random factor. The model also co-varied for sex, MoCA and BDI score. Post hoc independent sample *t* tests were used to assess for differences between groups and were corrected for multiple comparisons using the Bonferroni method.

Multiple regression analyses were used to correlate [^11^C]DTBZ BP_ND_ within each ROI against clinical measures including UPDRS-III score, Hoehn and Yahr score, LEDD amount, and disease duration while factoring patient group condition (i.e., PD patients with and without probable RBD). This regression model included sex, MoCA and BDI as co-variates. All tests were completed using SPSS (version 21; Chicago, IL); and the alpha level was set to 0.05 as a cut-off to determine significance.

## Results

The demographic, clinical and psychological characteristics for each group are summarized in Table 1. There were no differences between all participant groups (i.e., healthy controls, PD patients without probable RBD, and PD patients with probable RBD) for age, radiotracer injected dose, injected mass, and specific activity. PD patients with and without probable RBD were comparable in relation to their disease duration, UPDRS-III score, Hoehn and Yahr score, and LEDD amount. However, differences were observed between groups for sex, MoCA, and BDI. These three variables were included as co-variates in subsequent analyses. There were no statistical outliers using the 1.5 interquartile range method.

As there were main effects of MoCA [F_(2, 42)_ = 7.29, *p* = 0.002] and BDI [F_(2, 42)_ = 3.89, *p* = 0.028], we followed up with Bonferroni post hoc *t*-tests to detect where the group differences occurred. These particular results were reported previously by Valli et al. [24]. In summary for BDI, patients with probable RBD had the highest depression score, then PD patients without probable RBD, then healthy controls. Only PD patients with probable RBD were statistically higher than healthy controls (*t* = − 2.68, *p* = 0.03). Regarding MoCA, in comparison to healthy controls, PD patients with probable RBD had the lowest cognitive score (*t* = 3.70, *p* = 0.003), followed by patients without probable RBD (*t* = 2.66, *p* = 0.0165). PD patients with probable RBD had relatively lower MoCA score compared to PD patients without probable RBD but this difference did not reach significance.

**Fig. 1:**
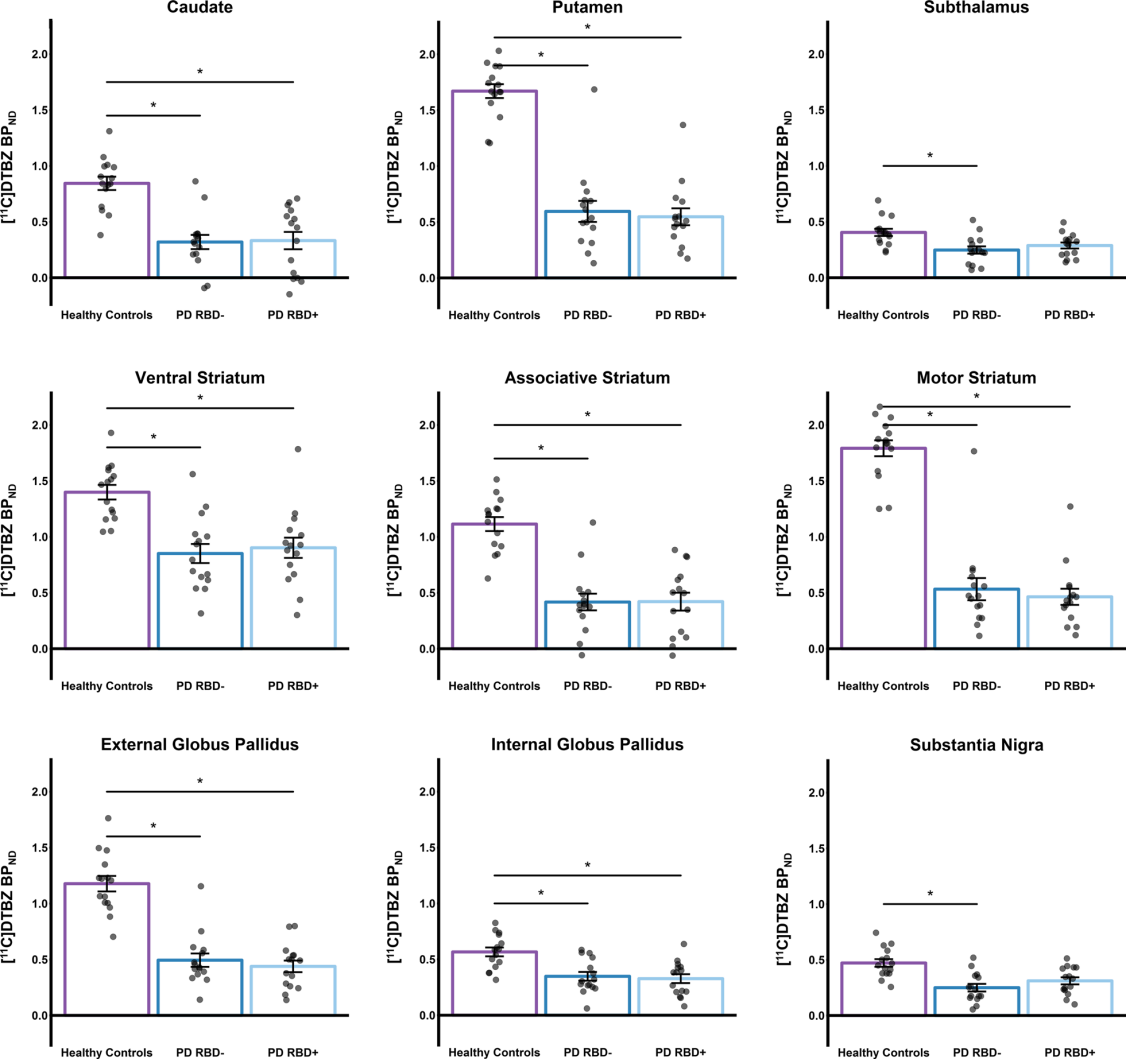
This figure displays the BP_ND_ differences between groups of healthy controls, PD patients without probable RBD (PD-RBD−), and PD patients with probable RBD (PD-RBD+) in all explored regions within the basal ganglia. All regions revealed to have significant main effect. [^11^C]DTBZ BP_ND_ represents the degree of VMAT2 availability. We found that BP_ND_ of PD-RBD− was reduced compared to healthy controls in all regions shown. This pattern was similarly seen for PD-RBD+ in relation to healthy controls for all regions except for the subthalamus and substantia nigra. *p < 0.05, Bonferroni corrected

Results from the mixed effect model, co-varying for sex, MoCA and BDI score, revealed significant main effect of group on [^11^C]DTBZ binding within all ROIs explored: caudate [F_(2, 39)_ = 10.6, *p* < 0.001], putamen [F_(2, 39)_ = 41.57, *p* < 0.001], associative striatum [F_(2, 39)_ = 17.49, *p* < 0.001], motor striatum [F_(2, 39)_ = 53.49, *p* < 0.001], ventral striatum [F_(2, 39)_ = 7.42, *p* = 0.002], external globus pallidus [F_(2, 39)_ = 29.17, *p* < 0.001], internal globus pallidus [F_(2, 39)_ = 7.33, *p* = 0.002], substantia nigra [F_(2, 39)_ = 7.58, *p* = 0.002], and subthalamus [F_(2, 39)_ = 4.61, *p* = 0.016]. Specifically, we found that the mean [^11^C]DTBZ BP_ND_ of PD patients without probable RBD were lower than healthy controls for all these significant ROIs. Similarly, PD patients with probable RBD had reduced BP_ND_ compared to healthy controls for all these significant ROIs except for the subthalamus and substantia nigra. Summary of the Bonferroni corrected post-hoc *t*-test results between PD patients without probable RBD versus healthy controls and PD patients with probable RBD versus healthy controls is displayed in Table 2. We were not able to detect statistical differences between PD patients with and without probable RBD for these significant regions (Fig. 1). In summary, the significant findings from the mixed effects model was driven mainly by the differences between healthy controls and both PD subgroups, not the differences between PD patients with and without probable RBD.

**Table 2:** Post-hoc *t*-test results looking at [^11^C]DTBZ BP_ND_ differences between PD-RBD− vs. healthy controls and PD-RBD+ vs. healthy controls

Basal ganglia ROIs	(A) PD-RBD− vs healthy controls		(B) PD-RBD+ vs healthy controls	
	*t* value	*p* value	*t* value	*p* value
Caudate	4.38	< 0.001	3.72	0.002
Putamen	8.44	< 0.001	7.76	0.001
Subthalamus	3.02	0.013	1.96	0.168
Ventral striatum	3.77	0.002	2.84	0.021
Associative striatum	5.59	< 0.001	4.84	< 0.001
Motor striatum	9.51	< 0.001	8.89	< 0.001
External globus pallidus	6.92	< 0.001	6.68	< 0.001
Internal globus pallidus	3.33	0.006	3.49	0.04
Substantia nigra	3.90	0.001	2.35	0.072

This table displays the Bonferroni corrected post-hoc *t*-test results for significant ROIs detected through the mixed effects model. Column A on the left displays results looking at [^11^C]DTBZ BP_ND_ differences between PD patients without probable RBD (PD-RBD−) and healthy controls. Column B on the right shows results between PD patients with probable RBD (PD-RBD+) and healthy controls

**Fig. 2:**
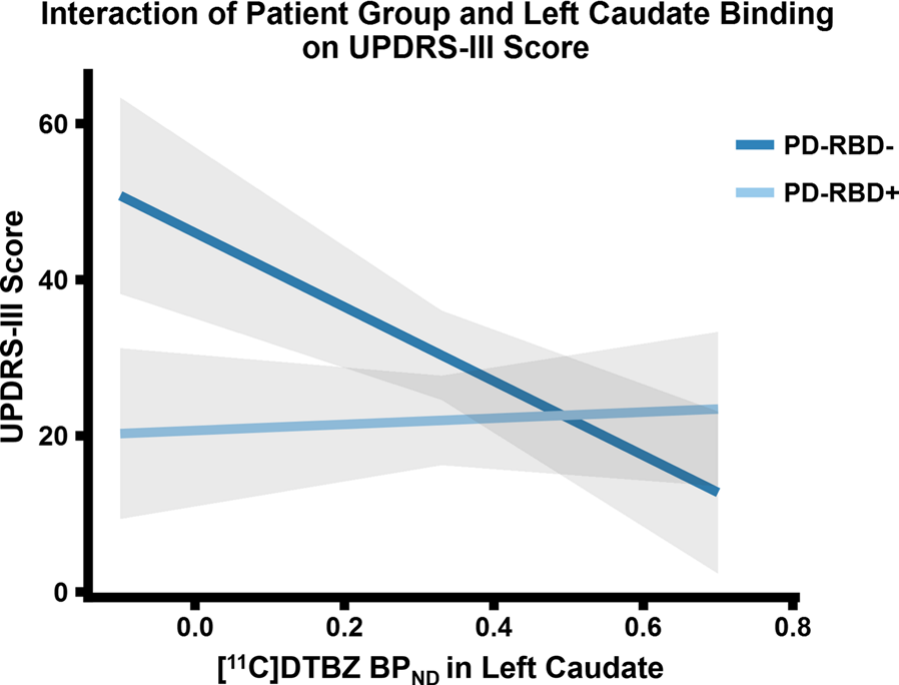
This interaction plot is a result from the regression analysis. Patient group moderates the left caudate BP_ND_ in PD patients without probable RBD (PD-RBD*−*): as the UPDRS-III score increases, PD-RBD*− *patients have lower VMAT2 availability. However, this relationship is non-existent for PD patients with probable RBD (PD-RBD+). This figure was plotted using marginal means that accounted for the included co-variates: sex, MoCA and BDI. The grey band for each line represents the 95% confidence interval

Multiple regressions were computed to predict clinical measures (i.e., disease duration, UPDRS-III score, Hoehn and Yahr score, and LEDD amount) from group condition (i.e., PD patients with and without probable RBD), and [^11^C]DTBZ BP_ND_ of all basal ganglia ROIs explored, while covarying for sex, MoCA and BDI score. We were able to observe a significant interaction between left caudate BP_ND_ and group condition where these two measures predicted UPDRS-III score (F_(1, 23)_ = 11.13, *p* = 0.003, R^2^ = 0.59). Figure 2 displays the interaction plot between left caudate BP_ND_ and UPDRS-III score where there is a steep negative correlation for PD patients without probable RBD. This relationship was not present for PD patients with probable RBD. No other significant interactions were detected through the multiple regression analyses between the BP_ND_ of the significant ROIs and group condition on clinical scores.

## Discussion

This study aimed to characterize whether PD patients with probable RBD had more extensive striatal dopamine denervation pathology relative to PD patients without RBD. We observed that both PD patient subgroups had lower tracer binding compared to healthy controls within the basal ganglia. There were no indications to show [^11^C]DTBZ binding differences between PD patients with and without probable RBD, which did not confirm our hypothesis. The reduced radioligand signal compared to healthy controls reflects lower VMAT2 availability in both patient subgroups—which implies nigrostriatal denervation as a result of PD neurodegeneration. In the group of PD patients without probable RBD, there was a strong negative relationship between left caudate VMAT2 availability and UPDRS-III score. In other words, with the worsening of motor severity in patients without probable RBD, it correlated with more presynaptic denervation in the left caudate. However, this relationship was not present in PD patients with probable RBD.

The observed reduction of VMAT2 density as measured by [^11^C]DTBZ in our PD patient sample in the putamen, caudate, substantia nigra, and globus pallidus relative to controls is consistent with previous neuroimaging studies of VMAT2 [8, 38–41]. This reinforces the notion that [^11^C]DTBZ PET imaging holds the potential to effectively differentiate PD patients from controls [42, 43]. The observed significant correlation between motor severity for PD patients and left caudate BP_ND_ was also consistent with previous studies in their sample of PD patients with no reports of other co-morbidities [42, 43]. This relationship is in line with the view that motor disability in PD is primarily associated with impairment of subcortical structures within the nigrostriatal pathway [44]. In contrast, no significant interactions were observed between radiotracer binding and clinical measures in PD patients with probable RBD, thus implying that other neurochemical abnormalities may account for this clinical complication.

Unlike previous DAT studies [15, 17, 45], we found no evidence of VMAT2 level differences between groups of PD patients with and without probable RBD. This observation is consistent with a previous investigation that used [^11^C]DTBZ PET imaging as a secondary focus to their study objectives in PD patients with and without probable RBD [46]. Specifically, they were unable to differentiate VMAT2 levels of PD patients with probable RBD from PD patients without probable RBD within the caudate and putamen [46]. Our current study was different from this previous study where we used age-matched healthy controls and explored more brain regions within the basal ganglia, beyond just the caudate and putamen. There are several possible reasons that could explain the lack of differences in VMAT2 levels between PD patients with and without probable RBD that we observed. VMAT2 has been demonstrated to be a stable marker for presynaptic nigrostriatal integrity as it is less prone to changes induced by medications or compensatory mechanisms associated with the loss of dopaminergic neurons [19]. Our findings reflect that VMAT2 remained unaffected by the co-morbidity of probable RBD in conjunction with presynaptic changes associated with medication or compensatory mechanisms that would normally occur in PD [19]. This implies that the PD pathophysiology is the primary driving force depleting VMAT2 availability in both groups.

An alternative explanation may involve the relationship between the activities and availability of VMAT2 and DAT in the presynaptic terminals [47]. Previous literature has consistently shown that PD patients with probable RBD have lower DAT availability relative to PD patients and controls [15, 17, 45]. This observed reduction of DAT levels would in turn lower the neural ability to reuptake dopamine back into the presynaptic terminals for vesicular repackaging and subsequent reutilization [18, 48, 49]. The lower levels of presynaptic vesicles with dopamine should in turn result in a reduction of the VMAT2 levels in PD with probable RBD. However, this rationale was defied by the lack of differences in VMAT2 levels between PD patients with and without probable RBD. It could be possible that there were varying endogenous intravesicular dopamine levels in PD patients with and without probable RBD that may have netted in negligible differences in BP_ND_ signalling between the two groups [18].

Previous literature shows other molecular structures and neurotransmitters that may play a larger role contributing to RBD in PD apart from DAT [15, 17, 45]. Outside the striatum, our group showed a negative relationship where D2 receptor availability within the uncus parahippocampus decreased with increasing disease severity in PD patients with probable RBD relative to PD patients without probable RBD [24]. Other studies showed altered cholinergic [46] and noradrenaline levels [50], along with changes in glucose metabolic activity [16] in PD patients with probable RBD relative to patients without probable RBD, suggesting RBD pathophysiology in PD is multi-systemic that impacts regions beyond the striatum and the dopaminergic system.

This study has some limitations to consider. Our patients were screened for probable RBD through the first question of the Mayo Sleep Questionnaire as part of their routine neurology clinic visit. These patients were not confirmed through the sleep polysomnography test. Despite this, the questionnaire has been validated in two studies. The first study validated the questionnaire against polysomnography in a multi-centre prospective cohort trial that included patients suspected to have neurodegenerative disease and healthy older adults. This study achieved a sensitivity of 98% and specificity of 74% [26]. A follow up validation study of the Mayo Sleep Questionnaire was carried out in a community-based sample of 128 participants who had underwent a previous polysomnography test, and resulted in a sensitivity of 100% and specificity of 95% [27]. These evidences makes the sleep questionnaire a useful research tool in settings where polysomnography is not readily available [51].

Another limitation is that patients were assessed on several clinical measures while “ON” dopamine replacement therapy and they were “OFF” medication during the PET scan. In turn, the relationship between motor severity and dopaminergic degeneration within the basal ganglia may not be fully representative even though VMAT2 was shown to be less prone to medication influences [38]. Although we showed a relationship between nigrostriatal innervation and motor score in PD patients without probable RBD, investigating motor features while “ON” and “OFF” medication and changes with VMAT2 levels may provide more insight about the true relationship observed between PD with and without probable RBD and motor severity in relation to the left caudate VMAT2 availability.

## Conclusion

In comparison to age-matched controls, the current study revealed that PD patients with and without probable RBD had lower VMAT2 levels within the subcortical structures of the basal ganglia which reflects denervation within the nigrostriatal pathway. No significant interactions were detected between the BP_ND_ and clinical scores in PD patients with probable RBD. These findings collectively suggest that VMAT2 and striatal dopamine denervation in general may not be a significant contributor to the pathophysiology of RBD in PD patients. Future studies are encouraged to explore other underlying neural chemistry mechanisms to better understand the driving force of RBD in PD patients.

## Data Availability

The datasets used and/or analysed during the current study are available from the corresponding author on reasonable request.
